# Interprofessional geriatric competencies in the DACH region (Germany, Austria and Switzerland): Insights from a multinational multilingual open online survey

**DOI:** 10.1007/s00391-026-02646-2

**Published:** 2026-09-07

**Authors:** M. Honegger, S. Lindner-Rabl, G. Ogliari, L. Soraci, Ch. Pertinatsch, M. C. Polidori, R. Roller-Wirnsberger

**Affiliations:** 1 Department of Internal Medicine, Research Unit Services for Old Age and Lifelong Health, Medical University of Graz, Auenbruggerplatz 15, 8036 Graz, Austria; 2 Department for Scientific Research in Rehabilitation, Pension Insurance Austria, Vienna, Austria; 3 Leicestershire County Council, National Institute for Health and Care Research (NIHR) Health Determinants Research Collaboration (HDRC) Leicestershire, County Hall, Leicester Road, LE3 8RA Glenfield, Leicestershire UK; 4 Unit of Geriatric Medicine, Italian National Research Center on Aging (IRCCS INRCA), Cosenza, Italy; 5 Ageing Clinical Research, Department II of Internal Medicine and Center for Molecular Medicine Cologne, Faculty of Medicine and University Hospital Cologne, Cologne, Germany; 6 Cologne Cluster of Excellence on Aging and Aging-associated Diseases (CECAD), University of Cologne, Cologne, Germany

**Keywords:** Geriatrics, Interprofessional education, Patient-centered care, Healthcare professionals, Europe, Geriatrie, Interprofessionelle Ausbildung, Patientenzentrierte Versorgung, Gesundheitsfachkräfte, Europa

## Abstract

**Background:**

Geriatric care requires coordinated, interprofessional and person-centered approaches; however, geriatric competencies among healthcare professionals (HCP) vary considerably across Europe, and only limited data are available for the DACH (D: Deutschland, Germany; A: Austria; CH: Confoederatio Helvetica, Switzerland) region.

**Objectives:**

To investigate self-perceived knowledge, relevance and interest regarding key geriatric topics and skills among HCPs in the DACH region and to compare findings with other European countries.

**Material and methods:**

Secondary data analyses of the PROGRAMMING COST Action 21122 online survey. The DACH responses (*n* = 291) were compared with those from other European countries (*n* = 5134) and 33 geriatric topics and skills were analyzed to identify educational priorities.

**Results:**

The HCPs working in the DACH region rated their perceived geriatric knowledge higher than HCPs working in other European countries. Nevertheless, substantial perceived knowledge gaps remained, particularly in the areas of dementia, depression, chronic pain, cognitive screening and sarcopenia. Perceived relevance and interest in further education exceeded self-assessed knowledge levels. In contrast, topics such as oncogeriatrics, gerodontology and geriatric emergency care were assigned lower priority.

**Conclusion:**

Despite overall high self-perceived competence, important gaps persist, especially in complex, interdisciplinary areas. Continuing education needs appear to be strongly influenced by clinical practice and system structures. Given the large number of medical doctors in our data, some implications are intraprofessional; nevertheless, targeted competency-based cross-professional educational initiatives and frameworks are needed to strengthen geriatric care and support workforce mobility across Europe.

**Supplementary Information:**

The online version of this article (https://doi.org/10.1007/s00391-026-02646-2) contains supplementary material, which is available to authorized users.

## Abstract (de)

**Hintergrund:**

Die geriatrische Versorgung erfordert koordinierte, interprofessionelle und personenzentrierte Ansätze. Die Kompetenzen von Gesundheitsfachkräften sind jedoch europaweit unterschiedlich, wobei für den DACH-Raum (Deutschland, Österreich, Schweiz) nur begrenzte Daten vorliegen.

**Ziel der Arbeit:**

Untersuchung des selbst eingeschätzten Wissens, der Relevanz sowie des Interesses im Bereich geriatrischer Themen und Kompetenzen bei Gesundheitsfachkräften im DACH-Raum sowie Vergleich mit anderen europäischen Ländern.

**Material und Methoden:**

Sekundäre Datenanalyse der Online-Befragung im Rahmen der PROGRAMMING COST-Action 21122. Die Antworten aus dem DACH-Raum (*n* = 291) wurden mit jenen aus anderen europäischen Ländern (*n* = 5134) verglichen. Dabei wurden 33 geriatrische Themen und Kompetenzen analysiert, um Fortbildungsschwerpunkte zu ermitteln.

**Ergebnisse:**

Gesundheitsfachkräfte, die im DACH-Raum arbeiten, schätzten ihre Kenntnisse höher ein als jene in anderen europäischen Ländern. Dennoch zeigten sich deutliche Wissenslücken, insbesondere in den Bereichen Demenz, Depression, chronische Schmerzen, kognitive Screenings und Sarkopenie. Die wahrgenommene Relevanz sowie das Interesse an Fortbildungen überstiegen das selbst eingeschätzte Wissen. Themen wie Onkogeriatrie, Gerodontologie und geriatrische Notfallversorgung wurden hingegen weniger prioritär bewertet.

**Diskussion:**

Trotz insgesamt hoher selbst eingeschätzter Kompetenz bestehen Defizite, insbesondere in komplexen, interdisziplinären Bereichen. Der Fortbildungsbedarf scheint maßgeblich von der klinischen Praxis und Systemstrukturen beeinflusst zu werden. Durch die hohe Ärztezahl in den Daten sind einige Schlussfolgerungen auf diese Gruppe begrenzt, gleichwohl sind gezielte berufsgruppenübergreifende Fortbildungsmaßnahmen und kompetenzbasierte Rahmenkonzepte erforderlich, um die geriatrische Versorgungsqualität zu stärken und die Mobilität von Fachkräften innerhalb Europas zu fördern.

Geriatric care requires coordinated, interprofessional, and person-centered approaches; however, competencies of healthcare professionals (HCPs) vary across Europe and data for the DACH region (Germany; Austria; Switzerland) remain scarce. This study reports on how HCPs working in the DACH region assess their self-perceived knowledge, relevance, and interest in key geriatric topics and skills, and their educational needs. It also compares the responses with those from other European countries, highlighting relevant gaps in practice.

## Background

A quality hallmark of geriatric care is coordinated, person-centered and integrated collaboration between healthcare professionals (HCP) [29, 32]. To ensure high-quality care, professionals must be adequately prepared to deliver geriatric care and work collaboratively [19, 25]; however, the geriatric competence of HCPs involved in team-based care could vary substantially across Europe. This is reflected in data from a recent open online survey conducted within the European PROGRAMMING (PROmoting GeRiAtric Medicine in countries where it is still eMergING) COST (European COoperation in Science and Technology) Action 21122, an international collaborative initiative aiming to strengthen geriatric medicine through education and training on basic geriatric concepts [20]. The survey assessed self-perceived competence of geriatric medicine and educational needs regarding key geriatric topics and skills among HCPs across the WHO region Europe and Kosovo*^1^, and other countries [17, 18].

To date, data on the knowledge, skills and attitudes of the geriatric workforce in the Germany-Austria-Switzerland (DACH) region are still limited. Therefore, this study examined the results of the survey regarding perceived knowledge, relevance and interest in key geriatric topics and skills. Furthermore, we compared the responses from HCPs working in the DACH region with those from HCPs working in other European region countries to identify specific educational needs within the DACH region and to explore potential DACH-specific educational priorities beyond general patterns.

## Study design and investigation methods

The current publication builds on data collected during the PROGRAMMING COST Action 21122 and was conducted in accordance with the Declaration of Helsinki and ethics approval was obtained when required by local regulations. All details of the survey development, structure, aim, content, translation, dissemination as well as first results have been previously published [8, 17, 18]. Statements on data availability, funding and details on ethical compliance are provided in Supplementary file 1. For the present subanalysis, responses from the DACH region from sections 1 (queries related to demographics) and 2 (self-rated knowledge, relevance and interest in further training on major geriatric topics and skills) of the survey were extracted to a separate database, analyzed and compared with responses from the other European region countries. Answers on 33 key geriatric topics and skills, including perceived knowledge, relevance and interest in the respective topics were analyzed.

The three domains were scored as follows: very low (1), low (2), fair (3), high (4) and very high (5). Furthermore, participants rated their overall perceived knowledge and competence in caring for older people. Possible responses ranged from very poor (1) to very good (5). We used descriptive statistics, Wilcoxon rank-sum test for DACH vs. other countries, ordinal logistic regression of overall perceived knowledge, adjusted for age and gender (odds ratios, OR, 95% confidence intervals, CI), global topic score (relevance + interest − knowledge) standardized to Z‑scores and quintiles. Further survey details as well as all details on the data analysis are provided in Supplementary File 2.

## Results

### Demographics

After excluding medical students and HCPs working outside European region countries, this study’s analytical sample included the responses of *n* = 291 HCPs working in the DACH region and *n* = 5134 HCPs working in other European region countries. Supplementary File 3 shows the demographics of participants.

The results showed that although Austria’s population is one tenth the size of Germany’s, *n* = 141 responses were received from Austria, compared to *n* = 131 responses from Germany and *n* = 19 feedbacks from Switzerland. As can be seen, respondents from the DACH region were older on a nonsignificant level compared to respondents from the remaining European region countries (mean age DACH 46.3 years, SD 11.9 years, other European countries 42.4 years, SD 11.9 years).

Medical doctors represented the largest group (40.2%) of respondents, followed by nurses (13.4%), professionals not directly in contact with patients (e.g. researchers, 8.9%), dieticians (6.9%) and occupational therapists (6.9%). Other roles included art therapists (4.1%), physiotherapists (3.1%), dentists (3.1%), pharmacists (2.8%), medical doctors in training (2.8%), educationalists (2.1%), nurse/healthcare assistants (1.7%), social workers (1.4%), psychologists/psychotherapists (1.0%). speech and language therapists (0.7%), dental technicians/hygienists (0.3%), podiatrists (0.3%) and other healthcare professionals (0.3%).

**Fig. 1:**
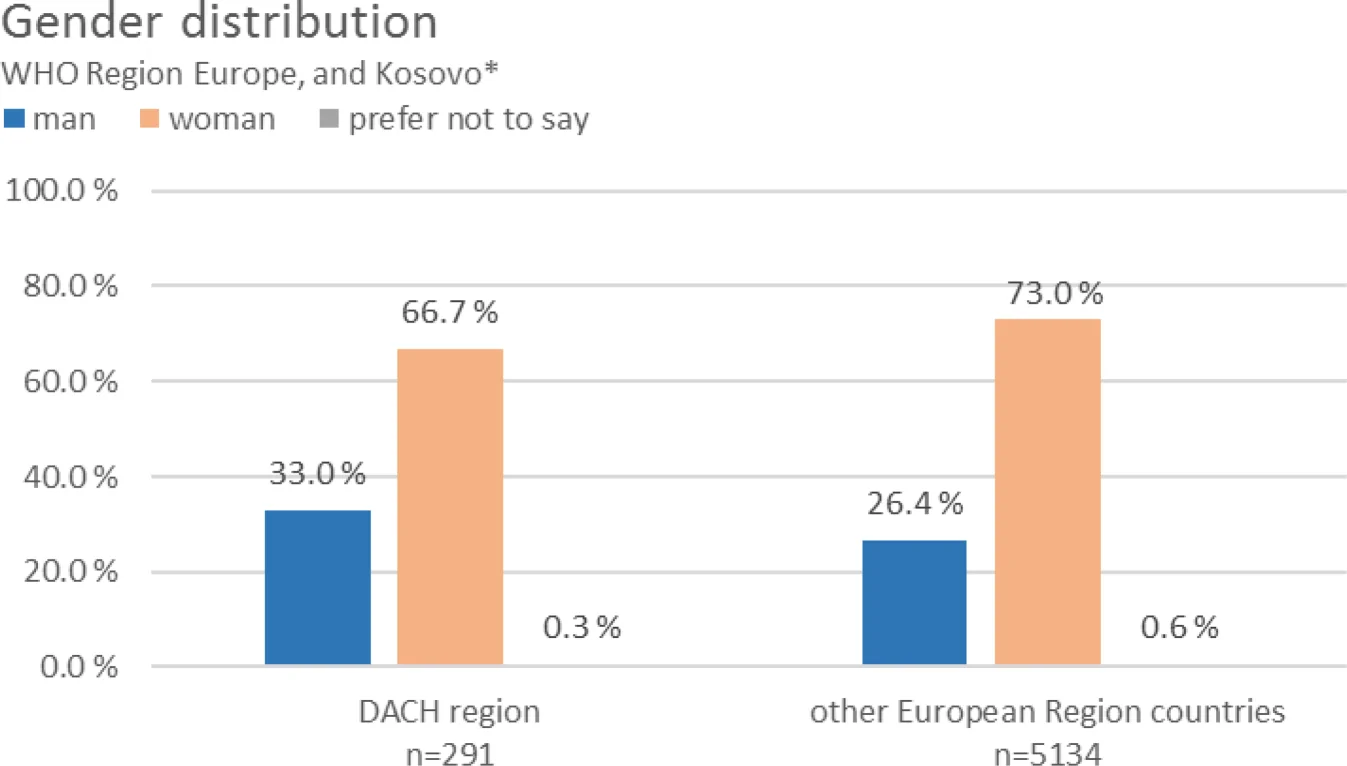
Gender distribution of healthcare professionals participating in the survey

As in other European countries, a large proportion of women (66.7%) responded to the survey in the DACH region (Fig. 1).

### Overall perceived knowledge and competence in the care for older people

**Fig. 2:**
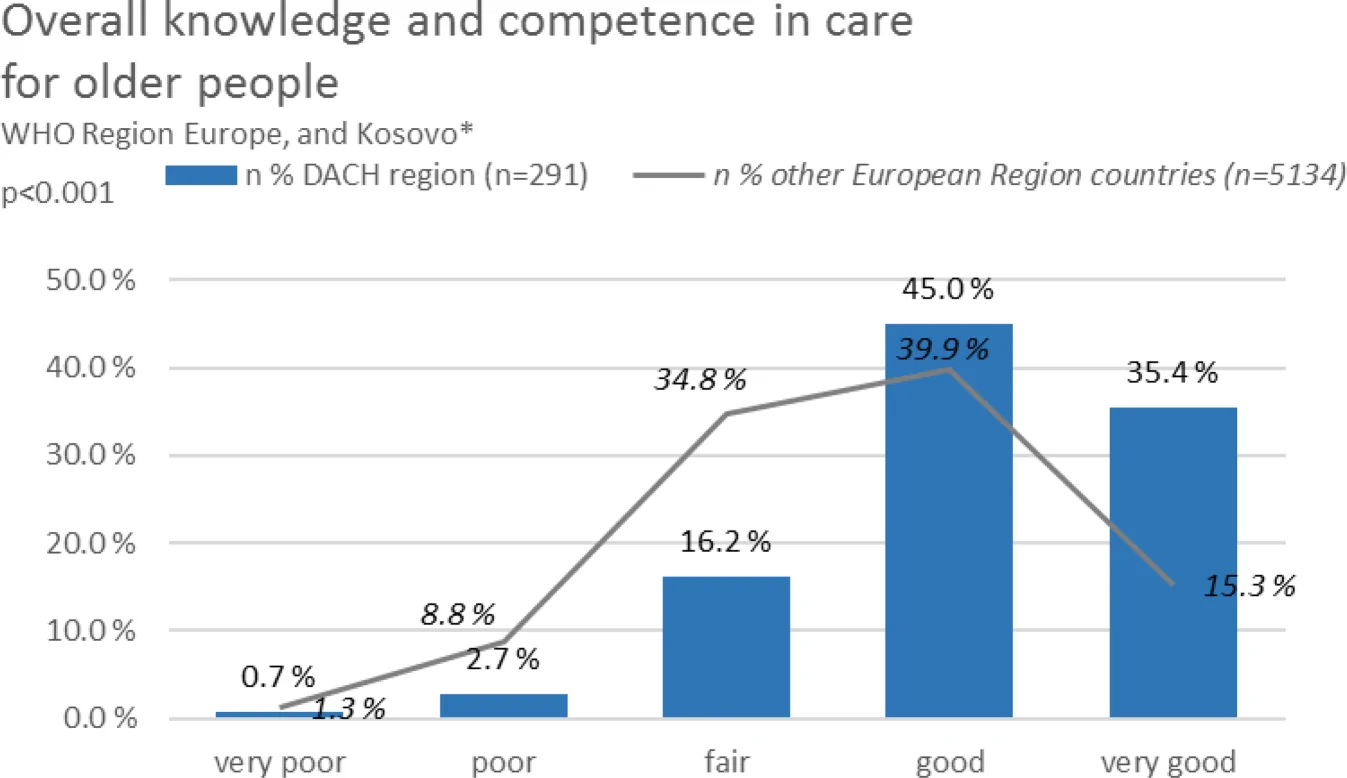
Overall perceived knowledge and competence in care for older people in the DACH region compared to the responses of the other European region countries. Numbers in italics refer to other European countries

Self-perceived knowledge in geriatrics was high among DACH HCPs, as shown in Fig. 2. Most respondents rated their knowledge as good (45.0%) or very good (35.4%), while only a small proportion reported poor (2.7%) or very poor (0.7%) knowledge. Compared with participants from other European region countries, DACH respondents reported significantly higher levels of self-assessed knowledge (*p* < 0.001).

This relationship was confirmed by age and gender-adjusted ordinal regression analysis, where HCPs from the DACH region had significantly higher odds of reporting greater overall perceived knowledge and competence in geriatric care compared with HCPs from other European region countries (OR 2.92, 95% CI 2.35–3.65), independently of age and gender. Older age was also independently associated with higher perceived knowledge and competence (OR 1.02 per additional year, 95% CI 1.02–1.03). In contrast, gender was not significantly associated with the outcome (OR 1.10, 95% CI 0.99–1.23).

### Topics and skills

**Fig. 3:**
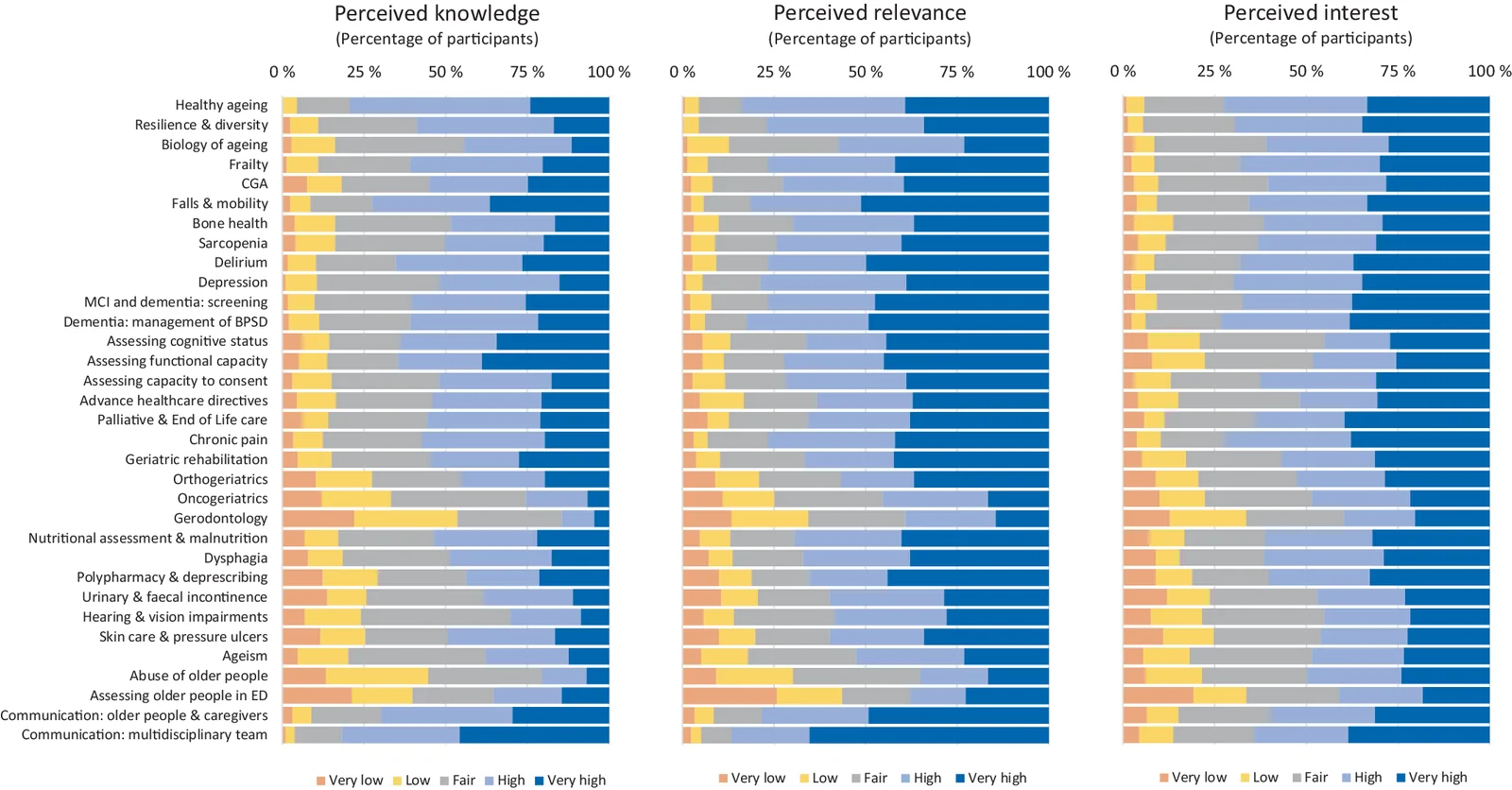
Diagrams of perceived knowledge (left), relevance (middle) and interest (right) in skills and topics in the DACH region ranging from very low to very high

To provide a more comprehensive overview, response distributions for all 33 questions from the DACH region regarding perceived knowledge, relevance and interest in topics and skills related to the care of older people were analyzed and are presented in Fig. 3.

A consistent pattern of relevance > interest > knowledge was observed across nearly all domains, indicating that DACH HCPs broadly perceived geriatric topics as clinically important and expressed motivation to learn beyond their current level of self-reported knowledge. The overall knowledge score was 4.12.

The highest knowledge scores across topics were recorded for communication within a multidisciplinary team (mean knowledge score 4.23), followed by healthy aging (3.99), falls and mobility (3.98), communication with older people and caregivers (3.88) and assessing functional status (3.84). Conversely, the lowest knowledge scores were observed for gerodontology (2.43), abuse of older people (2.69), oncogeriatrics (2.87) and assessing older people in the emergency department (ED) (2.89). Polypharmacy and deprescribing, urinary and fecal incontinence, and hearing and vision impairments also showed relatively low knowledge scores. More details are shown in Supplementary File 4.

### Comparison of DACH results to other European countries

Compared with other European region countries (Supplementary File 5), DACH HCPs reported higher perceived knowledge across almost all topics. Relevance ratings were similarly higher, while interest varied more. Areas of noteworthy difference (more than 20% deviation from other European region countries) were found for the topics healthy ageing (26.5%), falls and mobility (22.1%), delirium (24.5%), dementia (26.2%), assessment of cognitive status (25.0%), functional capacity (26.3%) and malnutrition (26.9%), advance healthcare directives (25.5%), palliative care (22.3%), chronic pain (24.1%), geriatric rehabilitation (27.8%), orthogeriatrics (21.3%) and dysphagia (22.3%). Results comparable between DACH respondents and the remaining survey participants from Europe were found for transversal skills, such as communication within the multidisciplinary team (27.8%) and with older adults and their caregivers (20.8%).

Results for perceived relevance underlined these scorings; however, results from the interest domain also included a few topics with negative deviation such as assessing cognitive status (−10.9%), indicating a lack of awareness for these topics in their daily business.

### Educational needs

**Fig. 4:**
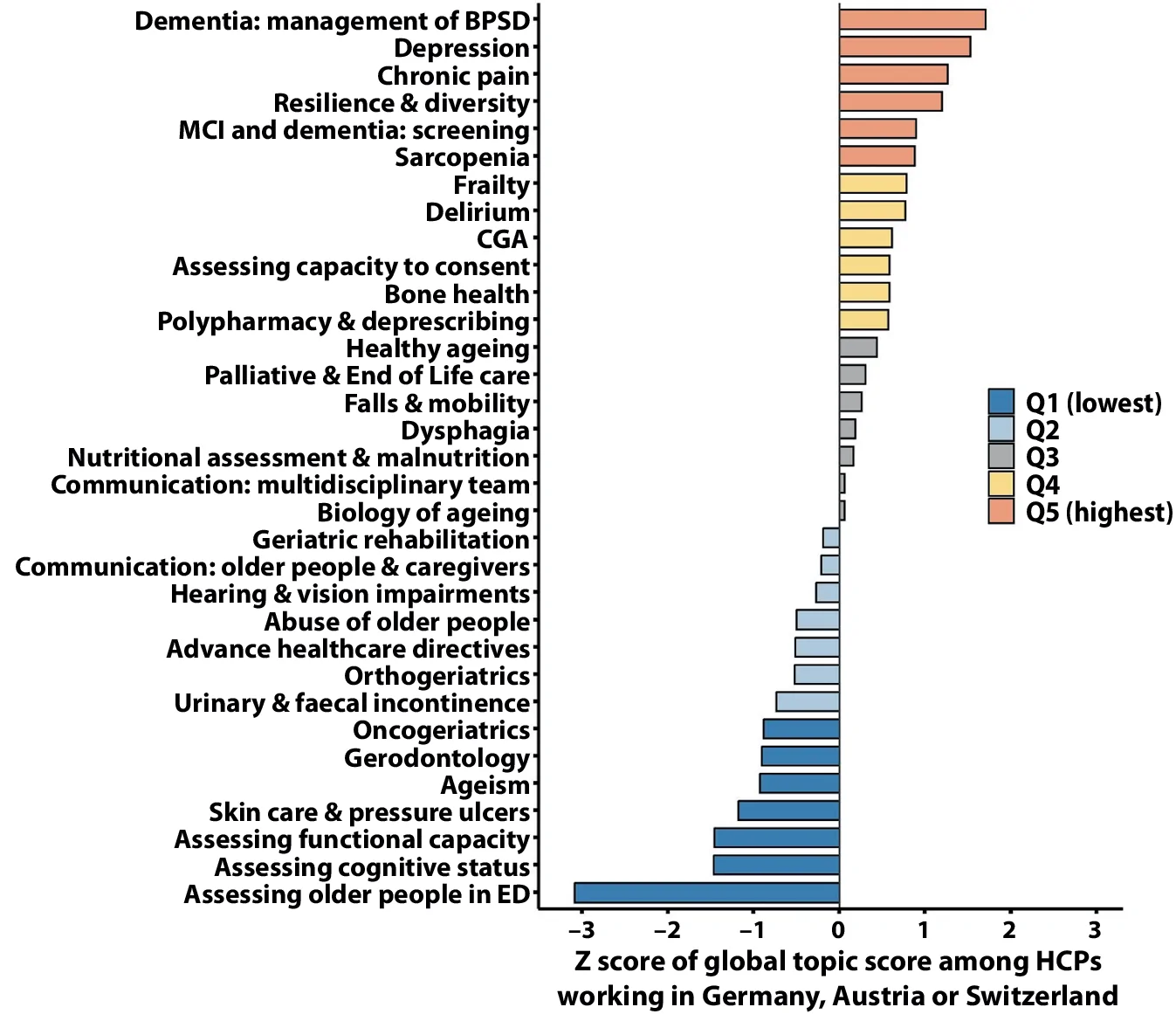
Z‑scores of global topic scores (interest + relevance − knowledge) in the DACH region (*n* = 291, ranked by quintile)

The Z‑scores of the global topic scores are presented in Fig. 4, summarizing the unmet educational needs. A positive Z‑score indicates topics where respondents reported high perceived relevance and interest but comparatively low knowledge, reflecting areas of high educational need. Management of behavioral and psychological symptoms of dementia (BPSD) showed the largest positive score (Z-score = 1.70), followed by depression (1.53), chronic pain (1.26), resilience and diversity (1.19), mild cognitive impairment (MCI) and dementia: screening (0.89) and sarcopenia (0.88), all falling in the highest quintile of the Z‑score distribution.

Negative Z‑scores, indicating domains where knowledge was relatively high compared to perceived relevance and interest, were observed for a cluster of topics in Q1 and Q2. Assessing older people in ED showed the most extreme negative Z‑score of −3.09, followed by assessing cognitive status (−1.47), assessing functional status (−1.46), skin care and pressure ulcers (−1.18), ageism (−0.92), gerodontology (−0.90) and oncogeriatrics (−0.88).

Overall, our findings indicate that DACH HCPs not only perceived themselves as more competent in geriatric care knowledge but also reported a higher perceived relevance for many topics than peers in other European region countries. Educational needs for HCPs seem to be highest in the topics of dementia-management, depression, chronic pain, resilience and diversity as well as sarcopenia.

## Discussion

This study provides insights into self-assessed geriatric competence, as well as the perceived relevance of and interest in geriatric topics among HCPs in the DACH region compared with other European region countries.

Key findings indicate that HCPs across the DACH region report higher self-perceived geriatric knowledge than respondents from other European countries. This may partly reflect the professional mix and seniority: DACH had a slightly higher share of medical doctors (42.9% vs. 39.3%) and among them a larger proportion were not in (postgraduate) training (93.6% vs. 72.5%, DACH *n* = 117 not in training vs. *n* = 8 in training, other countries *n* = 1462 not in training vs. *n* = 554 in training). Geriatric topics are included in all the national undergraduate medical curricula in the DACH region [13, 15, 16, 22] and the competence level of medical students is likely comparable across the countries at the time of graduation. All three countries include basic knowledge and skills transfer for geriatric competencies during postgraduate training curricula. Taken together, greater clinical seniority and daily contact with older people may shape professional capacity, while interprofessional geriatric education further enhances skills, knowledge and quality of care [14]. Given that 59.8% of respondents were non-medical HCPs, our data indicate interprofessional priorities in high-need topics (e.g. dementia, depression, chronic pain, MCI screening, sarcopenia) and in interprofessional communication, consistent with the variety of continuous professional development (CPD) offers present for HCPs [27]. In Germany and Austria, postgraduate CPD is based on accredited continuing education programs as well as master’s programs, with the aim to foster the development of professional workplace competence [1]. This finding underpins the importance of CPD programs already present and raises the need for a structured and evidence-based development of educational offers for HCPs already working with older people independent of the setting.

The question on how to design these programs can be answered by the results of this publication, at least content-wise. The Z‑score data (Fig. 4) demonstrate a clear educational need in the topics of management of dementia, depression, chronic pain, resilience and diversity, screening for MCI and dementia, as well as sarcopenia. This is in line with the literature and epidemiological data demonstrating a high prevalence and disease burden of these conditions among older adults [2, 5].

In addition, the care of older adults is challenged by the high prevalence of multimorbidity, frailty and increasing care dependency, all of which contribute to the complexity of clinical decision-making, requiring integrated, personalized and interprofessional approaches [3, 10, 21, 25, 26, 31]. In this context, preventive strategies play a crucial role, particularly tertiary prevention focusing on the maintenance of independence and the prevention of geriatric syndromes, as well as quaternary prevention aiming to reduce overtreatment [4, 24].

In contrast, several topics showed negative Z‑scores, albeit for different reasons. Some areas, such as assessing functional capacity, are characterized by high perceived knowledge, with mean values that exceed those of most other topics as well as the perceived relevance and interest attributed to the same content. This suggests educational attainment rather than disengagement. Other areas, including oncogeriatrics, gerodontology and assessment in the ED, combine low perceived knowledge with even lower perceived relevance and interest, indicating limited structural integration within the healthcare system. This distinction is important, as it highlights how structural and organizational conditions shape HCPs capacities within the system. Unlike orthogeriatrics, which is well established and widely implemented, oncogeriatrics and gerodontology have not yet gained comparable recognition or structural integration in the DACH region. This hypothesis that professional capacity building is strongly influenced by environmental factors, is supported by literature emphasizing that professional competencies are shaped through the interaction of healthcare systems, educational structures, and local population needs. [9]. Our analysis found a similar picture for geriatric skills in emergency care. Although several EDs across the DACH region have already implemented screening procedures for older patients based on tools such as the clinical frailty scale [23], structures, geriatric processes and standard operating procedures do not align with evidence-based concepts of senior-friendly EDs [28]. It is likely that this situation also affects HCPs’ awareness of the topic.

Interestingly, one of the most highly ranked transversal skills by HCPs in the DACH region was interprofessional communication. This capacity seems the key to work in geriatric teams as it builds the cornerstone for goal-oriented care planning, respectful collaboration and sustainable patient security [30]. These findings clearly set the demand for a continued education based on the concept of interprofessional education (IPE). Such IPE standards have been set in the US-IPEC Framework, by the Irish colleagues in the HSE Framework, and others [11, 12] and could serve as best practice examples for design of future educational offers.

One of the main goals of the PROGRAMMING COST Action 21122 is to develop a European interprofessional educational framework addressing regional training needs, which can guide future CPD programs across all professions engaged in geriatric care. The observed differences between the DACH region and other European countries also open up opportunities for increased international exchange whilst using this new framework as the basic standard for CPD programs. Common curricula, cross-border continuing education initiatives, and standardized competency frameworks could help to harmonize the quality of geriatric care across Europe and strengthen interprofessional collaboration. This appears particularly relevant in the light of demographic change and increasing mobility of HCPs. Competency-based frameworks could enable transparent recognition of skills and support targeted upskilling pathways for migrating professionals, thereby helping to address workforce shortages and variability in training standards [6, 7].

When interpreting the results, it is important to consider that cultural and systemic factors could have influenced self-assessment. Differences in professional role models, training structures, and healthcare systems could limit comparability across countries. Furthermore, greater awareness of geriatric issues in the DACH region could also lead to a more nuanced evaluation of individual competency areas.

### Strengths and limitations

A key strength of the study is its multiprofessional sample from 3 countries, providing a broad perspective on geriatric care. The combined assessment of perceived knowledge, relevance and interest enables a nuanced analysis of educational needs.

Nevertheless, several limitations apply: reliance on self-reported data, heterogeneity across HCP groups with a large proportion of medical doctors in the DACH subsample (thus interprofessional generalizations should be cautious), potential selection bias due to uneven country participation, and female overrepresentation, which may limit generalizability. Findings should also be interpreted in the light of cross-country differences in education pathways and CPD frameworks, particularly among non-medical HCPs.

## Practical recommendations

The HCPs in the DACH region report high levels of self-perceived knowledge in geriatric care; however, the analysis identified substantial educational needs, particularly in complex, multidisciplinary conditions such as dementia-related symptoms, depression, chronic pain and sarcopenia.

These findings appear to be influenced by formal training, professional background and clinical exposure, with higher training levels linked to greater perceived competence, especially among physicians.

At the same time, topics considered highly relevant seem to reflect how HCPs encounter these conditions in daily practice, whereas the prioritization of other topics could be shaped by existing structural and organizational frameworks within healthcare systems.

These insights are highly relevant for developing targeted CPD programs and could contribute to competency-based frameworks aimed at supporting workforce mobility and defining minimum requirements for HCPs entering the DACH region.

## Supplementary Information

ESM1: Supplementary material 1

ESM2: Supplementary material 2

ESM3: Supplementary material 3

ESM4: Supplementary material 4

ESM5: Supplementary material 5

## Data Availability

The datasets generated by the survey can be made available upon request by following the appropriate procedure. Any member of PROGRAMMING can propose research or policy questions to be explored within the survey data following a specific set of recommendations on how to access them. A review committee will assess each proposal and decide whether access to the data should be granted. We encourage every member of PROGRAMMING to submit a specific and structured proposal form to the review committee, which will supervise and coordinate the various proposals, to promote cooperation and prevent duplication of efforts. New applications to join the European Cooperation in Science and Technology Action PROGRAMMING CA21122 are possible for the entire duration of the action and the process for this it outlined on the Action website (“COST. European COoperation in Science and Technology. COST Association. URL: https://www.cost.eu/” and “PROGRAMMING—PROmoting GeRiAtric Medicine in countries where it is still eMergING homepage. PROGRAMMING. URL: https://cost-programming.eu/”).

## Footnotes

1 * Even though Kosovo is not included in the official list of WHO European Region countries, for the sake of our analysis we included data coming from this country under the general category “European Region countries” (encompassing WHO European Region countries + Kosovo), given the participation of Kosovo in the COST Action. This designation is without prejudice to positions on status, and is in line with UNSCR 1244/1999 and the ICJ Opinion on the Kosovo declaration of independence.

## Declarations

PROGRAMMING COST Action 21122 was conducted in accordance with the Declaration of Helsinki and ethical approval was obtained. The study was performed in accordance with the ethical standards as laid down in the 1964 Declaration of Helsinki and its later amendments. Ethics approval was obtained in the countries where local regulations necessitated review by an ethics committee, as previously detailed (Ogliari G, Masud T, Herghelegiu AM, et al. Educational Needs in Geriatric Medicine Among Health Care Professionals and Medical Students in COST Action 21122 PROGRAMMING: Mixed-Methods Survey Protocol. JMIR Res Protoc. 2025;14:e64985. Published 2025 Jun 3. https://doi.org/10.2196/64985) Informed consent was electronically obtained by each participant at the beginning of the survey. Participants were informed of the EuGMS privacy policy in compliance with the General Data Protection Regulation (GDPR; EU 2016/679).
