# Supplementary material for: Interprofessional geriatric competencies in the DACH region (Germany, Austria and Switzerland): Insights from a multinational multilingual open online survey
Source: Z Gerontol Geriatr. 2026 Sep 7;59(6):515–21. doi: 10.1007/s00391-026-02646-2 (PMC13597566; doi:10.1007/s00391-026-02646-2)
Supplement: Supplementary file 1 [file 391_2026_2646_MOESM4_ESM.docx]

**Supplementary File 4**

Mean knowledge, relevance, and interest scores across all 33 geriatric topics among HCPs in DACH countries (Germany, Austria, Switzerland; n=291)
