# Supplementary material for: Interprofessional geriatric competencies in the DACH region (Germany, Austria and Switzerland): Insights from a multinational multilingual open online survey
Source: Z Gerontol Geriatr. 2026 Sep 7;59(6):515–21. doi: 10.1007/s00391-026-02646-2 (PMC13597566; doi:10.1007/s00391-026-02646-2)
Supplement: Supplementary file 2 [file 391_2026_2646_MOESM5_ESM.docx]

**Supplementary File 5**

Perceived knowledge, relevance and interest in skills and topics in other European Region countries ranging from very low to very high.
