# Supplementary material for: Interprofessional geriatric competencies in the DACH region (Germany, Austria and Switzerland): Insights from a multinational multilingual open online survey
Source: Z Gerontol Geriatr. 2026 Sep 7;59(6):515–21. doi: 10.1007/s00391-026-02646-2 (PMC13597566; doi:10.1007/s00391-026-02646-2)
Supplement: Supplementary file 3 [file 391_2026_2646_MOESM1_ESM.doc]

**Supplementary File 1**

Funding

This research received no external funding. However, this study is realised in the context of PROGRAMMING COST Action 21122, funded by the European Cooperation in Science and Technology under grant agreements No. AGA-CA21122-1-15351, -2-16937, -3-17032 and -4-18281. The funder had no role in the study design, data collection and analysis, or manuscript preparation.

Data availability

The datasets generated by the survey may be made available upon request by following the appropriate procedure. Any member of PROGRAMMING can propose research or policy questions to be explored within the survey data following a specific set of recommendations on how to access them. A review committee will assess each proposal and decide whether access to the data should be granted. We encourage every member of PROGRAMMING to submit a specific and structured proposal form to the review committee, which will supervise and coordinate the various proposals, to promote cooperation and prevent duplication of efforts. New applications to join the European Cooperation in Science and Technology Action PROGRAMMING CA21122 are possible for the entire duration of the Action, and the process for this it outlined on the Action website [“COST. European COoperation in Science and Technology. COST Association. URL: https://www.cost.eu/” and “PROGRAMMING - PROmoting GeRiAtric Medicine in countries where it is still eMergING homepage. PROGRAMMING. URL: <https://cost-programming.eu/>”].

Compliance with ethical standards

The study was performed in accordance with the ethical standards as laid down in the 1964 Declaration of Helsinki and its later amendments. Ethics approval was obtained in the countries where local regulations necessitated review by an ethics committee, as previously detailed [Ogliari G, Masud T, Herghelegiu AM, et al. Educational Needs in Geriatric Medicine Among Health Care Professionals and Medical Students in COST Action 21122 PROGRAMMING: Mixed-Methods Survey Protocol. JMIR Res Protoc. 2025;14:e64985. Published 2025 Jun 3. doi:10.2196/64985]. Informed consent was electronically obtained by each participant at the beginning of the survey. Participants were informed of the EuGMS privacy policy in compliance with the General Data Protection Regulation (GDPR; EU 2016/679).

Recent publications from the COST-Action Programming

Ogliari, G., Kotsani, M., Benetos, A. *et al.* Perceived educational needs in geriatric medicine of professionals: a multinational multilingual open online survey. *Eur Geriatr Med* (2026). <https://doi.org/10.1007/s41999-026-01456-4>.

Ogliari G, Masud T, Herghelegiu AM, Pavic T, Wissendorff Ekdahl A, Piotrowicz K, Duque S, Benetos A, Bogdanovic N, Bonin-Guillaume S, Frost R, Koca M, Kossioni A, Kravvariti E, Martínez-Velilla N, McKeown W, Roller-Wirnsberger R, Savas S, Vassallo M, Yellon T, Petrovic M, Kotsani M; Collaborators of the PROGRAMMING survey on educational needs. Educational Needs in Geriatric Medicine Among Health Care Professionals and Medical Students in COST Action 21122 PROGRAMMING: Mixed-Methods Survey Protocol. JMIR Res Protoc. 2025 Jun 3;14:e64985. doi: 10.2196/64985. PMID: 40305653; PMCID: PMC12174867.

Harasani, K., Ogliari, G., Soraci, L. *et al.* Pharmacists’ perceived knowledge, attitudes and practice in geriatrics: findings from a multinational multilingual open online survey. *Eur Geriatr Med* (2026). <https://doi.org/10.1007/s41999-026-01504-z>

Preliminary data on the survey were further presented at the “Top 10 Value-Based Geriatric Care Models and Interventions: Towards a Sustainable Healthcare System, Improving Older Adults’ Quality of Life” joint event by the PROGRAMMING COST Action CA21122 and the Ordem dos Medicos (the Order of Physicians of Portugal), Lisbon, Portugal, on April 17 to 19, 2024; the European College of Gerodontology 34th Annual Congress: New Frontiers in Gerodontology in Belgrade, Serbia, on June 6 to 7, 2024; the European Society of Cardiology Congress 2024 in London, United Kingdom, on August 30 to September 2, 2024; during the Presentation of the preliminary results of the big Pan-European survey of PROGRAMMING COST Action open session at the 20th European Geriatric Medicine Society Congress in Valencia, Spain, on September 19, 2024; during the “Joining efforts to develop geriatric medicine across Europe – the experience of EU-funded projects” joint webinar with other European projects on ageing (PROGRAMMING CA21122, ICARE 4 OLD (Individualized CARE for Older Persons with Complex Chronic Conditions at home and in nursing homes) project, and AFFIRMO [Atrial Fibrillation Integrated Approach] project) on July 2, 2024; during the “Orthogeriatrics – a model of care towards improving outcomes in older patients with hip fracture and promoting Geriatric Medicine” training webinar organized by PROGRAMMING CA21122 in collaboration with the Fragility Fracture Network on October 24, 2024; at the conference within the framework of COST Action 21122 PROGRAMMING entitled “Optimal care models for older adults: bridging community care and Geriatric Medicine in Krakow, Poland”, on January 23 and 24, 2025; in the abstract “PROmoting GeRiAtric Medicine In Countries Where It Is Still EMergING (PROGRAMMING) CA21122: The Survey on Educational Needs” at the 20th European Geriatric Medicine Society Congress in Valencia, Spain, on September 18 to 20, 2024; at the PROGRAMMING COST Action 21122 and SPRIN-G Joint Event “Real-life challenges in the continuity of care for older adults” in Heraklion, Greece, June 16-18, 2025; in the oral presentation “Key gaps in geriatric knowledge and educational needs among healthcare professionals working with older adults in acute, subacute, and long-term care settings across Europe: a COST PROGRAMMING 21122 survey analysis across Europe and beyond”, in the poster “European medical students’ attitudes to becoming geriatricians—data from COST PROGRAMMING action CA 21122” at the 21st Congress of the European Geriatric Medicine Society, Reykjavik, Iceland, September 24–26, 2025.
