# Supplementary material for: Interprofessional geriatric competencies in the DACH region (Germany, Austria and Switzerland): Insights from a multinational multilingual open online survey
Source: Z Gerontol Geriatr. 2026 Sep 7;59(6):515–21. doi: 10.1007/s00391-026-02646-2 (PMC13597566; doi:10.1007/s00391-026-02646-2)
Supplement: Supplementary file 4 [file 391_2026_2646_MOESM2_ESM.doc]

**Supplementary File 2**

**Details on Study design and investigation methods**

Survey details

The survey was co-created by a core group of experts in the care of older adults from various professional backgrounds and several countries and finalised after review from an external panel of international stakeholders (Ogliari et al., 2025). It was originally created in English and then translated into 24 other languages (including German, Italian and French) through a rigorous and centrally-coordinated translation process. The survey was launched under the umbrella of national and international geriatric and other scientific societies and health and educational organisms’ contacts to promote participation from as many different professions as possible involved in the care for older people. All participants provided informed consent electronically at the beginning of the survey and were informed about **European Geriatric Medicine Society (EuGMS)** privacy policy in compliance with the General Data Protection Regulation (GDPR; EU 2016/679). Ethical approval was obtained according to local regulations (Ogliari et al., 2025).

The survey content comprised 8 sections and a wide range of questions including detailed queries related to demographics (section 1), self-rated knowledge, relevance and interest in further training on major geriatric topics and skills (section 2) and country of current work (section 4) (Ogliari et al., 2026).

The datasets generated by the survey may be made available to PROGRAMMING members upon request by following the appropriate procedure as outlined (see data availability).

Data analysis

Quantitative analysis were performed using SPSS Version 28 (https://www.ibm.com/support/pages/downloading-ibm-spss-statistics-29). Data visualisation was generated using GraphPad Prism version 9.0 (https://www.graphpad.com/updates/prism-900-release-notes) and MS Excel Professional Plus 2016. Descriptive statistics was used to summarise participant characteristics and survey responses. Professional categories were grouped for reporting purposes, with several original survey categories combined (e.g. “other professionals” for roles not directly in contact with patients). Categorical variables were presented as frequencies and percentages, whereas continuous variables were presented as mean and standard deviation (SD).

To compare overall perceived knowledge and competence between HCPs working in the DACH region and those working in other European Region countries, the Wilcoxon rank-sum test was applied. To further evaluate whether the association between DACH membership and overall perceived knowledge remained significant after adjustment for potential confounders, multivariable ordinal logistic regression analyses were performed. Overall perceived knowledge was entered as the dependent variable, while DACH membership was included as the main exposure variable and age and gender as covariates. Results are presented as odds ratios (ORs) with 95% confidence intervals (95% CIs). The comparison between the DACH region and other European countries was based on descriptive frequency comparisons to identify noteworthy differences (>20%).

Furthermore, to measure educational needs for each of the 33 topics and skills we calculated the mean (SD) knowledge score, mean relevance score and mean interest score, respectively, and ranked the topics and skills by descending mean scores. To link together the three dimensions of perceived knowledge, relevance, and interest, we applied the following formula to the responses of each individual participant as described by Ogliari et al., previously: *Global topic score = perceived relevance score + perceived interest score – perceived knowledge score.* This score prioritises topics with high relevance, high interest and low knowledge. To standardise this global topic score and enhance the comparability of educational needs across topics, we computed Z-scores for each global topic score. For example, the Z-score for CGA was computed as: *Z-score for CGA = [(CGA global topic score) – (the mean of all 33 global topic scores)] / (SD of all 33 global topic scores).* Consequently*,* topics were ranked according to their Z-scores and categorised into quintiles (Q1-Q5), each representing 20% of the distribution.

References:
