# Supplementary material for: Interprofessional geriatric competencies in the DACH region (Germany, Austria and Switzerland): Insights from a multinational multilingual open online survey
Source: Z Gerontol Geriatr. 2026 Sep 7;59(6):515–21. doi: 10.1007/s00391-026-02646-2 (PMC13597566; doi:10.1007/s00391-026-02646-2)
Supplement: Supplementary file 5 [file 391_2026_2646_MOESM3_ESM.doc]

**Supplementary File 3**

Demographics of survey participants

| **Demographics** | **DACH region** | **other European Region countries** |
| --- | --- | --- |
| **Total number** | **n=291** | **n=5134** |
| *Austria* | 141 (48,45%) | - |
| *Germany* | 131 (45,02%) | - |
| *Switzerland* | 19 (6,53%) | - |
| **Age, (mean (SD))** | 46.3 (SD 11.9) | 42.4 (SD 11.9) |
| **Role or profession, n (%)** |  |  |
| *Art therapist* | 12 (4.12%) | 16 (0.31%) |
| *Dental technician or hygienist* | 1 (0.34%) | 36 (0.7%) |
| *Dentist* | 9 (3.09%) | 495 (9.64%) |
| *Dietician* | 20 (6.87%) | 158 (3.08%) |
| *Educationalist* | 6 (2.06%) | 92 (1.79%) |
| *Medical Doctor in training (post-graduate)* | 8 (2.75%) | 554 (10.79%) |
| *Medical doctor (not in training)* | 117 (40.21%) | 1461 (28.46%) |
| *Nurse* | 39 (13.4%) | 805 (15.68%) |
| *Nurse assistant or healthcare assistant* | 5 (1.72%) | 34 (0.66%) |
| *Occupational therapist* | 20 (6.87%) | 106 (2.06%) |
| *Pharmacist* | 8 (2.75%) | 335 (6.53%) |
| *Physiotherapist* | 9 (3.09%) | 330 (6.43%) |
| *Podiatrist / chiropodist* | 1 (0.34%) | 2 (0.04%) |
| *Psychologist or psychotherapist* | 3 (1.03%) | 142 (2.77%) |
| *Radiographer* | 0 (0%) | 6 (0.12%) |
| *Social worker* | 4 (1.37%) | 86 (1.68%) |
| *Speech and language therapist* | 2 (0.69%) | 35 (0.68%) |
| *Other healthcare professionals* | 1 (0.34%) | 148 (2.88%) |
| *Other professionals (not directly in contact with patients such as researcher, healthcare manager, policymaker)* | 26 (8.93%) | 293 (5.71%) |

**Legend: The Table** shows the demographics and grouped professional categories of survey participants from the DACH region compared to other European Region countries.
